# Diagnostic Accuracy of Maximum Intensity Projection in Diagnosis of Malignant Pulmonary Nodules

**DOI:** 10.7759/cureus.6120

**Published:** 2019-11-11

**Authors:** Naila Jabeen, Ruby Qureshi, Amjad Sattar, Musarat Baloch

**Affiliations:** 1 Radiology, Dow University of Health Sciences, Karachi, PAK; 2 Internal Medicine, Liaquat University of Medical and Health Sciences, Hyderabad/Jamshoro, PAK

**Keywords:** maximum intensity projection, diagnostic accuracy, pulmonary nodule, sensitivity, specificity

## Abstract

Introduction

Pulmonary nodules are frequently encountered during chest imaging, and its evaluation is usually done by chest radiograph and computed tomography (CT) scan of chest. High resolution of multidetector CT (MDCT) has improved the nodule detection. Post processing techniques such as maximum intensity projection (MIP) can further improve the sensitivity of MDCT for nodule detection. Failure to diagnose malignancy in pulmonary nodules can delay the treatment. Therefore, the aim of this study was to determine the diagnostic accuracy of MIP in the diagnosis of malignant pulmonary nodules taking histopathology findings as gold standard.

Materials and methods

A retrospective cross-sectional study was conducted at Dow Institute of Radiology, Dow University of Health Sciences, from 1 December 2018 till 30 June 2019. Both male and female patients aged 18 years and above who underwent CT scan of chest with suspicion of pulmonary nodules were included. Patients already diagnosed with malignant pulmonary nodules and presenting for follow-up were excluded. Contrast-enhanced CT chest was performed on a multi-slice scanner. MIP reconstruction and evaluation was performed on the workstation. Sensitivity, specificity, positive predictive value, negative predictive value, and diagnostic accuracy of MIP were calculated taking histopathology findings as gold standard.

Results

A total of 202 patients were included in this study. The mean age of the patients was 55.87 ± 13.08 years. A total of 103 patients (51.0%) were males and 99 patients (49.0%) were females. There were 131 (64.9%) nodules with smooth margins and 71 (35.1%) nodules with irregular margins. The mean size of nodule was 3.1 ± 0.7 cm. Sensitivity, specificity, positive predictive value, negative predictive value, and diagnostic accuracy of MIP in diagnosing malignant pulmonary nodules were found to be 85.82%, 82.35%, 90.55%, 74.67%, and 84.65%, respectively, taking histopathology findings as gold standard. The nodules >3 cm in size had a higher sensitivity for diagnosing malignant pulmonary nodules. Smooth margin nodule had high sensitivity, specificity, and diagnostic accuracy for diagnosing malignant pulmonary nodules.

Conclusion

MIP images have high sensitivity, specificity, and diagnostic accuracy in the diagnosis of malignant pulmonary nodules. The utilization of MIP images can aid in the detection of malignant pulmonary nodules and help in formulating early treatment strategies for the patients. Other post processing techniques such as volume rendering and computer-aided detection can help in further improving patient care.

## Introduction

Pulmonary nodule is a frequent finding on thoracic imaging. Its evaluation is a challenge for diagnostic modalities [1,2]. Pulmonary nodules are frequently evaluated by chest radiograph. Computed tomography (CT) scans are also routinely used for characterization of pulmonary nodules. Detection rate by radiograph is low as compared to CT scans [3]. While comparing features of malignancy in pulmonary nodules, a study showed that 20.5% nodules have malignant features on CT scan, whereas 9.8% nodules showing malignant features on chest radiograph [4]. Radiotracer localization can also aid in intraoperative localization of pulmonary nodules [5].

The identification and diagnosis of pulmonary nodules has been revolutionized by multidetector CT (MDCT). MDCT has high resolution, and it improves nodule detection [6]. Post processing techniques also have a potential for further characterization of pulmonary nodule. A post processing technique known as maximum intensity projection (MIP) utilizes highest attenuating voxels projecting through the data set to form the final image [7]. Another post processing technique termed as volume rendering (VR) can also help in pulmonary nodule evaluation [7]. MIP can also be used in the detection of peritoneal metastatic disease [8].

The radiologist has the advantage of adjusting the slice thickness in the post processing techniques for accurate evaluation of pulmonary nodules. MIP has a sensitivity of 84% in diagnosing pulmonary nodules [7]. Differentiation of a benign from malignant solitary pulmonary nodule is important as their prognosis and management is completely different. Approximately 20% of the solitary pulmonary nodules are malignant [4], and therefore it is important to diagnose these nodules early. Failure to diagnose malignancy early can lead to spread of disease thereby increasing morbidity and mortality. The aim of this study was to determine the diagnostic accuracy of MIP in the diagnosis of malignant pulmonary nodules taking histopathology findings as gold standard.

## Materials and methods

This was a retrospective cross-sectional study conducted at Dow Institute of Radiology, Dow University of Health Sciences, from 1 December 2018 till 30 June 2019. Both male and female patients aged 18 years and above presenting with cough, hemoptysis, and weight loss and who underwent CT scan of chest with suspicion of pulmonary nodules were included. Patients already diagnosed with malignant pulmonary nodules and presenting for follow-up were excluded. On MIP, malignant solitary pulmonary nodule was defined as a nodule of 1 cm or more in size having irregular margins. Informed consent was taken from each patient before enrolling in the study. Contrast-enhanced CT chest was performed on a multi-slice scanner. MIP reconstruction was performed on the workstation. The MIP images were evaluated and reported by a radiologist having more than three years of experience in reporting chest CT scans. Biopsy of the suspicious pulmonary nodule was performed, and sample was sent for histopathology analysis. Data were analyzed using Statistical Package for Social Sciences (SPSS) version 20 (IBM Corp., Armonk, NY). Quantitative variables such as age, size of nodules, and number of nodules were mentioned as mean and standard deviation. Qualitative variables such as gender, margins of pulmonary nodules, and findings on MIP and histopathology were mentioned as frequency and percentage. Sensitivity, specificity, positive predictive value, negative predictive value, and diagnostic accuracy of MIP were calculated using contingency tables taking histopathology findings as gold standard. Stratification was done on the basis of age, gender, size of nodules, and margins of nodules, and sensitivity, specificity, positive predictive value, negative predictive value, and diagnostic accuracy were also calculated after stratification.

## Results

A total of 202 patients were included in this study. The mean age of the patients was 55.87 ± 13.08 years. There were 82 (40.6%) patients with ≤50 years of age and 120 (59.4%) with >50 years of age. A total of 103 patients (51.0%) were males and 99 patients (49.0%) were females. There were 131 (64.9%) nodules with smooth margins and 71 (35.1%) nodules with irregular margins. The mean size of nodule was 3.1 ± 0.7 cm. The baseline characteristics of the patients are summarized in Table 1.

**Table 1:** Baseline characteristics of the patients

Baseline characteristics of the patients (n = 202)		
	n	%
Age, years	55.87 ± 13.08^ǂ^	
≤50 years	82	40.6
>50 years	120	59.4
Gender		
Males	103	51.0
Females	99	49.0
Size of nodules, cm	3.1 ± 0.7^ǂ^	
≤3 cm	86	42.6
>3 cm	116	57.4
Number of nodules		
1	164	81.2
>1	38	18.8
^ǂ^Mean± SD, n: number		

Malignant pulmonary nodules on MIP were found in 127 (62.9%) of the patients, while on histopathology malignant pulmonary nodules were found in 134 (66.3%) of the patients (Table 2).

**Table 2:** MIP and histopathology findings MIP = maximum intensity projection

MIP and histopathology findings (n = 202)			
MIP findings	Histopathology findings		Total
	Positive	Negative	
Positive	115	12	127
Negative	19	56	75
Total	134	68	202

Sensitivity, specificity, positive predictive value, negative predictive value, and diagnostic accuracy of MIP in diagnosing malignant pulmonary nodules were found to be 85.82%, 82.35%, 90.55%, 74.67%, and 84.65%, respectively, taking histopathology findings as gold standard. Baseline characteristics such as age and gender were stratified, and after stratification sensitivity, specificity, positive predictive value, negative predictive value, and diagnostic accuracy are shown in Table 3.

**Table 3:** Stratification according to baseline characteristics PPV = positive predictive value, NPV = negative predictive value

Stratification according to baseline characteristics (n = 202)				
	Age		Gender	
	≤50 years	>50 years	Male	Female
Sensitivity	82.76%	88.16%	88.24%	83.33%
Specificity	83.33%	81.82%	77.14%	87.88%
PPV	92.31%	89.33%	88.24%	93.22%
NPV	66.67%	80.00%	77.14%	72.50%
Diagnostic accuracy	82.93%	85.83%	84.47%	84.85%

Stratification was also done with respect to size of nodules and margins of nodules, and the results are shown in Table 4.

**Table 4:** Stratification according to size and margins of nodules PPV = positive predictive value, NPV = negative predictive value

Stratification according to size and margins of nodules (n = 202)				
	Size of nodule		Margins of nodule	
	≤3 cm	>3 cm	Smooth	Irregular
Sensitivity	83.33%	87.84%	86.05%	85.42%
Specificity	88.46%	78.57%	88.89%	69.57%
PPV	94.34%	87.84%	93.67%	85.42%
NPV	69.70%	78.57%	76.92%	69.57%
Diagnostic accuracy	84.88%	84.48%	87.02%	80.28%

## Discussion

Pulmonary nodules have diverse etiology and may have malignant potential. Due to an increasing use of CT scan, its diagnosis is becoming challenging [9]. Pulmonary nodules have a long list of differential diagnoses, among them granuloma and lung cancer form a major part [10]. In daily routine clinical practice, CT scan with thin collimation has a high spatial resolution and reduced partial volume effect. This can help in detecting small pulmonary nodules [11]. However, one drawback includes the increased number of images that are required to be examined by the radiologist. Moreover, thin sections of CT images make it harder to differentiate between a lung nodule and a vessel.

Post processing imaging technique such as MIP has also shown an improvement in detection rates of pulmonary nodules [12]. The MIP imaging technique produces a single image that is a projection of highest density of the image encountered by X-ray beam when it is traversing a stack of certain images that usually are preselected images having a variable depth [13]. The MIP technique is based on the principal of keeping the object with the highest density per slice only [14]. Although there is some loss of depth information on MIP images, there are some advantages of this post processing technique. MIP can detect small nodules that are not usually detected by conventional axial images. Moreover, MIP enables better identification of small pulmonary nodules by reducing the number of images required for evaluation. This is due better traceability of intrapulmonary vascular structures, making nodules adjacent to them easy to percept. The study has shown that in comparison with thin-section axial MDCT, axial MIP images enhance the accuracy of detecting small pulmonary nodules [15].

In the current study, we determined the diagnostic accuracy of MIP images in the diagnosis of malignant pulmonary nodules. The results of our study showed that MIP has a high sensitivity and specificity in diagnosing malignant pulmonary nodules. This sensitivity is slightly higher to the one reported by other authors [14]. Another international study has demonstrated an increase in solitary pulmonary nodule detection by the use of MIP images in patients who underwent resection of metastases [15]. They also utilized computer-aided detection (CAD) for improving sensitivity of nodule detection.

Our study results have demonstrated that sensitivity was higher in patients aged more than 50 years and also in male patients, whereas the specificity was higher in female patients and in patients aged less than or equal to 50 years. Nodules greater than 3 cm in size had a higher sensitivity, whereas nodules having less than or equal to 3 cm had a high specificity in diagnosing malignant pulmonary nodules. However, the diagnostic accuracy was almost similar. Sensitivity, specificity, and diagnostic accuracy for smooth nodule margin were higher as compared to irregular nodule margin for diagnosing malignant pulmonary nodules.

Our study results have shown that MIP has a high positive predictive value and moderate negative predictive value for malignant pulmonary nodule detection. Another study has shown a high positive predictive value for diagnosing pulmonary nodules.

This study is not without certain limitations. One limitation of our study was that it was a single institute study. Another limitation was that interobserver variability was not calculated. Various studies have shown that variability does exist among the readers when labeling pulmonary nodules on MIP images. Therefore, interobserver variability should be calculated. Moreover, intraobserver variability was also not evaluated in our study.

It is recommended that studies should be carried out on a larger sample size for the evaluation of MIP in diagnosing malignant pulmonary nodules. Pulmonary nodules are also frequently seen in pediatric population, and this accuracy of MIP in this population should also be studied. Moreover, other post processing techniques such as VR and CAD should also be utilized for accurate detection of malignancy in a solitary pulmonary nodule. International studies have utilized MIP for the detection of peritoneal metastases [8]. Therefore, it is also recommended that further studies utilizing MIP for the evaluation of peritoneal metastases can be carried out in our population so that bulk of peritoneal disease can be effectively evaluated.

## Conclusions

MIP images have high sensitivity, specificity, and diagnostic accuracy in the diagnosis of malignant pulmonary nodules. The utilization of MIP images can aid in the detection of malignant pulmonary nodules and help in formulating early treatment strategies for the patients. Other post processing techniques such as VR and CAD can help in further improving patient care.
